# Rich Club Characteristics of Alcohol-Naïve Functional Brain Networks Predict Future Drinking Phenotypes in Rhesus Macaques

**DOI:** 10.3389/fnbeh.2021.673151

**Published:** 2021-06-02

**Authors:** Jared A. Rowland, Jennifer R. Stapleton-Kotloski, Greg E. Alberto, April T. Davenport, Phillip M. Epperly, Dwayne W. Godwin, James B. Daunais

**Affiliations:** ^1^Research and Academic Affairs Service Line, Mid-Atlantic Mental Illness Research Education and Clinical Center, Salisbury VA Medical Center, Salisbury, NC, United States; ^2^Department of Neurobiology and Anatomy, Wake Forest School of Medicine, Winston-Salem, NC, United States; ^3^Department of Psychiatry and Behavioral Medicine, Wake Forest School of Medicine, Winston-Salem, NC, United States; ^4^Department of Neurology, Wake Forest School of Medicine, Winston-Salem, NC, United States; ^5^Department of Physiology and Pharmacology, Wake Forest School of Medicine, Winston-Salem, NC, United States

**Keywords:** magnetoencephalography, substance use disorder, risk factor, primate, brain function

## Abstract

**Purpose**: A fundamental question for Alcohol use disorder (AUD) is how and when naïve brain networks are reorganized in response to alcohol consumption. The current study aimed to determine the progression of alcohol’s effect on functional brain networks during transition from the naïve state to chronic consumption.

**Procedures**: Resting-state brain networks of six female rhesus macaque (*Macaca mulatta)* monkeys were acquired using magnetoencephalography (MEG) prior to alcohol exposure and after free-access to alcohol using a well-established model of chronic heavy alcohol consumption. Functional brain network metrics were derived at each time point.

**Results**: The average connection frequency (*p* < 0.024) and membership of the Rich Club (*p* < 0.022) changed significantly over time. Metrics describing network topology remained relatively stable from baseline to free-access drinking. The minimum degree of the Rich Club prior to alcohol exposure was significantly predictive of future free-access drinking (*r* = −0.88, *p* < 0.001).

**Conclusions**: Results suggest naïve brain network characteristics may be used to predict future alcohol consumption, and that alcohol consumption alters functional brain networks, shifting hubs and Rich Club membership away from previous regions in a non-systematic manner. Further work to refine these relationships may lead to the identification of a high-risk drinking phenotype.

## Introduction

Alcohol use disorder (AUD) constitutes a global problem and is ranked among the top substance abuse problems in the United States, with over 70% of adults that struggle with substance use disorder estimated to abuse alcohol (Substance Abuse and Mental Health Services Administration (SAMHSA), 2020). AUD impacts global brain functional networks including the default mode, executive, attentional, salience and reward networks (Loeber et al., 2009; Park et al., 2010; Chanraud et al., 2011, 2013; Camchong et al., 2013; Sullivan et al., 2013) but the neurocircuitry underlying vulnerability and resilience to AUD is not clearly understood, making it difficult to establish viable, targeted treatment options.

This lack of clarity is due, in part, to the difficulty in capturing an alcohol-naïve baseline in human subjects. Clinical studies are often conducted with long-term drinkers at different drinking phases after changes in brain networks have already manifested. It is clear that AUD is characterized in part by dysfunctional information processing (Sullivan et al., 2000) that occurs in part through altered brain activity during both resting state (RS) and task performance in alcoholics (Rangaswamy and Porjesz, 2008) as compared to other neurological conditions (Georgopoulos et al., 2007). Functional brain networks including the default mode, salience, and executive networks are known to be sensitive to chronic alcohol use (Sullivan and Pfefferbaum, 2005; Sullivan et al., 2013; Weiland et al., 2014; Fede et al., 2019) however, the temporal nature and anatomic directionality of changes that occur remains unclear.

Studies such as IMAGEN have attempted to address this issue by evaluating adolescents prior to initiation of substance use (Maričić et al., 2020). Ivanov et al. (2020) used the IMAGEN study data to demonstrate that expected reductions in impulsivity were associated with lower levels of new onset drinking in an adolescent sample. They also found that blunted medial orbitofrontal activity in response to reward was associated with increased new onset and use. Nees et al. (2012) used the IMAGEN dataset to demonstrate that personality factors (novelty seeking, impulsivity, extraversion, and sensation seeking) were more strongly related to the initiation of drinking behaviors than reward related brain function, but that reward related brain function (particularly ventral striatum) was more strongly related to the development of problematic alcohol use. Heinrich et al. (2016) extended these findings in the IMAGEN study by including genetic factors. Results replicated the strong influence of personality factors on early initiation of drinking behaviors, while genetic factors appeared to become equally important when predicting future alcohol misuse behaviors. Reward related brain function did not contribute significant variance to the model. Similarly, Harper et al. (2019) used data from the Minnesota Twin Family Study Enrichment Sample to demonstrate that P3 amplitude mid-frontal theta power during an oddball task were predictors of new onset alcohol behaviors 3 years later in a sample of 14-year-old adolescents. While these studies offer valuable insight into the factors associated with initiation of use and onset of problematic use, they do not fully alleviate the confounding influence of environmental variables.

Non-human primate (NHP) models are valuable tools to help address the limitations of studies involving human participants (Grant et al., 2008). The model applied in the current study has been used to demonstrate that daily drinking for 15 months causes functional and genomic changes across the brain when contrasted against the alcohol naïve brain (Budygin et al., 2003; Floyd et al., 2004; Alexander et al., 2006; Carden et al., 2006; Anderson et al., 2007; Acosta et al., 2010; Cuzon Carlson et al., 2011; Mohr et al., 2013), as well as reorganization of brain networks measured by fMRI (Telesford et al., 2015) and significantly altered signal power of multiple bandwidths across the brain using magnetoencephalography (MEG; Rowland et al., 2017a). Using the same NHP model, alcohol naïve predictors of future drinking have also been identified. These include low cognitive flexibility (Shnitko et al., 2019), early drinking phenotypes (i.e., gulping vs. sipping; Grant et al., 2008; Baker et al., 2017), age, latency to begin drinking, and the number of “bouts” of drinking (Helms et al., 2014; Baker et al., 2017). No studies have examined alcohol naïve aspects of brain function as predictors of future drinking levels using this model.

The objective of the current study was a longitudinal examination of the trajectory of these changes at an earlier time point (6 months of chronic heavy alcohol intake) than previously examined (15 months chronic heavy alcohol intake) to identify at what point they begin to manifest and if baseline, alcohol-naïve indicators of future drinking can be identified.

## Materials and Methods

### Animals

Adult female rhesus monkeys (*n* = 6, 5–7 years old at study start) were subjects in an ongoing ethanol (EtOH) self-administration study. This age group reflects late adolescence to early adulthood in humans. The monkeys were trained on an operant panel to self-administer all fluids and food using a well-established drinking model that parallels levels and patterns of intake observed in alcoholics (Grant et al., 2008; Baker et al., 2014). This process begins with EtOH-naïve monkeys that are induced to drink escalating doses of EtOH (0.5, 1.0 and 1.5 g/kg) for 30 days at each dose (induction phase). This phase introduces the monkeys to the reinforcing properties of alcohol and results in rapid and equal daily alcohol intake without causing taste aversion. All monkeys were maintained at 1.5 g/kg for 20 drinking days while operant panels were serviced and re-programed. Animals were then provided free access to EtOH and water for 22 h per day, 5 days per week for 180 days. Sessions began at 11:00 am each day. MEG recordings were acquired under EtOH naïve conditions (Baseline) and after 180 open access drinking days (Free Access) to determine the impact of chronic, daily intake on RS brain function. The alcohol-naïve baseline served as a within-animal control dataset.

### Preparation for MEG Scans

Animals were fasted overnight from food but not EtOH prior to scans. Average time between last drink and sedation for imaging was 344.2 min (*SD* = 377.9, min = 0.0, max = 977) at the Free Access Scan. These time frames raise the possibility of acute withdrawal (Winger and Woods, 1973); however, symptoms of withdrawal were not observed during similar time frames on non-imaging days (Pieper and Skeen, 1977) and the anesthetic agent (propofol, a GABAA receptor positive allosteric modulator; Shin et al., 2018) helped ensure acute withdrawal symptoms were not present during data acquisition. Previous work has shown that acute withdrawal in this model peaks between 24–72 h (Cuzon Carlson et al., 2011), which is beyond the duration since the last drink present here. Animals were sedated with ketamine (12 mg/kg, i.m.) for transport to the MEG suite. Anesthesia was induced with a bolus injection of 2.0–4.0 mg/kg propofol to allow intubation and was maintained *via* intravenous continuous infusion of 200 μg/kg/min propofol *via* syringe pump (Sage, Orion Research Corporation, Cambridge, MA, USA). Animals were placed in a supine position and artificially ventilated. These preparations are consistent with our previous reports (Telesford et al., 2015; Rowland et al., 2017a).

### MEG Signal Recordings

Data were acquired using a whole head CTF Systems Inc. MEG 2005 neuromagnetometer system equipped with 275 first-order axial gradiometer coils. Head localization was achieved using a conventional three-point fiducial system (nasion and preauricular points). Each monkey was tattooed at each fiducial location to ensure consistent placement over time. Resting-state recording was conducted with animals lying supine for 5 min. Data were sampled at 1,200 or 2,400 Hz over a DC-300 or DC-600 Hz bandwidth, respectively. MEG data were preprocessed using synthetic 3rd order gradient balancing, whole trial DC offsetting, and band pass filtered from DC-80 Hz with powerline filtering. Data were visually inspected for obvious muscle artifact, and such epochs, if present, were discarded from further analyses. Following initial MEG recording, a T1 weighted MRI image was obtained for each animal for co-registration and localization of MEG signals.

### Network Analysis

Network analysis was conducted identically to previous work (Rowland et al., 2017a,b, 2018). Network analysis proceeded by first identifying nodes of the network and quantifying communication among those nodes. The resulting matrices are conducive to the application of graph theory for calculating metrics describing the topology of the network.

### Network Creation

#### Node Identification

For each animal 41 non-adjacent bilateral regions of interest (ROIs, voxel size = 2 × 2 × 2 mm) were identified in native brain space representing the default mode and reward networks. These networks have been previously demonstrated to be affected by chronic heavy alcohol consumption in humans (Chanraud et al., 2011; Müller-Oehring et al., 2015; Zhang and Volkow, 2019) and shown to be present in NHPs (Vincent et al., 2007; Mantini et al., 2011; Belcher et al., 2013). Brain regions included the anterior cingulate, medial and lateral orbital frontal cortex, principle sulcus, nucleus accumbens, caudate head and body, head of the putamen, parietal area, precuneus, lateral and medial amygdala, anterior, medial, and posterior hippocampus, vermis, anterior and posterior lobes of the cerebellum, thalamus, and anterior insula.

#### Functional Connectivity

Source series representing the unique weighted sum of the output across all MEG sensors for a specific ROI in the brain were calculated using a well-validated beamformer (synthetic aperture magnetometry, SAM; Robinson and Vrba, 1999; Hillebrand et al., 2005). Prior work has demonstrated the sensitivity of MEG beamformers to superficial as well as deeper sources (Stapleton-Kotloski et al., 2014, 2018). The weighted phase lag index (wPLI; Vinck et al., 2011) was calculated between all pairs of source series to establish functional connectivity, filtered between 1 and 80 Hz. The wPLI is a phase-based metric insensitive to fluctuations in source amplitude. A surrogate distribution of 5,000 unique pairs of phase-randomized time series was created for each animal individually (Prichard and Theiler, 1994). Connectivity was operationalized at the frequency with the greatest difference in wPLI value between the real and surrogate data, calculated as standard deviations. This approach allows the frequency at which connections occur to vary from connection to connection, representing a better model of brain activity than restricting connectivity to a specific frequency band (Chen et al., 2008; Hillebrand et al., 2012). To remove connections not different from noise, connections without a real-surrogate difference exceeding 2.5 standard deviations were left unconnected. The resulting networks were then thresholded by satisfying the equation S = log(N)/log(K) where N represents the number of nodes in the network and K the average degree using S = 2.5 (Hayasaka and Laurienti, 2010).

### Network Metrics

Network metrics calculated are listed in Table 2. Metrics were selected with a focus on characterizing the topology of the overall network. *Clustering Coefficient* was selected as an indicator of clustering and subgroup formation within the network. This metric was calculated as defined in Stam and Reijneveld (2007). *Modularity* was selected as an indicator of well-defined subnetworks within the larger network. This metric was calculated using the Louvain method of community detection (Blondel et al., 2008). The analysis was run 500 times, using the average number of modules (*Number Modules*) as outcome variables. *Assortativity* *coefficient* represents the correlation coefficient of the degree of nodes on each end of a connection. The degree of a node is the number of direct connections that node has to other nodes in the network. A positive coefficient suggests nodes are preferentially connecting to other nodes of similar degree, while a negative coefficient suggests nodes preferentially connect to those of different degree (Newman, 2002). Rich Club was selected as an indicator of the presence of a “network backbone.” The Rich Club is a subset of highly connected and highly interconnected nodes forming the basis of the broader network. Rich Club characteristics (Colizza et al., 2006) were calculated using 500 independently generated random networks. The number of nodes (*Rich Club Nodes*) within the Rich Club, the minimum degree of those nodes (*Rich Club Degree*), and interconnectivity among those nodes (*Rich Club Coefficient*) were used as outcome variables. The Rich Club Coefficient was weighted by the average of the same metric across the 500 random networks, representing the level of increased interconnectivity over a random network. The *Mean Connection Frequency* was calculated as the average of the frequency at which connections occurred across all connections in the network. The number of connections occurring in each of the canonical frequency bands (e.g., delta, theta, alpha, beta, gamma) was also calculated as outcome variables. *Hubs* of the network were identified as the 10% of nodes (*n* = 4) with the highest degree.

**Table 1:** Amount of EtOH consumed by subjects during the Free-Access Period in grams/kilogram.

Subject	Free access
1	3.8
2	4.4
3	5.4
4	5.5
5	3.3
6	5.9

*Note. n = 6. EtOH = ethanol*.

**Table 2:** Descriptive statistics of network metrics prior to and following exposure to ethanol.

Network metric	Baseline	Free access
Clustering coefficient	0.35 (0.1)	0.30 (0.1)
Global efficiency	0.59 (0.05)	0.56 (0.05)
Assortativity coefficient	−0.40 (0.1)	−0.19 (0.2)
Rich club coefficient	1.84 (0.1)	1.91 (0.3)
Rich club nodes	13.17 (1.7)	10.83 (4.3)
Rich club minimum degree	7.50 (1.1)	9.50 (2.7)
Number of modules	10.50 (3.4)	8.83 (4.4)
Mean connection frequency^a^	6.57 (5.7)	16.01 (10.6)
Delta connections	172.67 (96.0)	99.0 (99.7)
Theta connections	15.33 (25.4)	34.67 (45.4)
Alpha connections	4.00 (9.8)	15.33 (23.2)
Beta connections	68.33 (106.9)	58.33 (47.9)
Gamma connections	1.67 (4.1)	54.67 (64.5)

*Data are presented as mean (standard deviation). Note. *n* = 6. ^a^Paired samples *t*-test between EtOH Naïve Baseline and Free-Access drinking *p* < 0.05; EtOH = ethanol*.

### Materials

Beamforming and source series construction were completed using software provided by CTF MEG International Services LP (Coquitlam, BC, Canada). Further analyses of source series data and network creation were conducted using Matlab 2016a. Network metrics were calculated using the Brain Connectivity Toolbox (Rubinov and Sporns, 2010). SAS Enterprise Guide 7.1 (SAS Institute Inc., Cary, NC, USA) was used for statistical analysis.

### Analyses

Differences across time in network metrics (Baseline to Free Access) were examined using paired samples *t*-tests. Consistency over time in the distribution of hub and Rich Club members was examined using paired samples *t*-tests. The data used are presented in Tables s 4, 5. The independent variable was time point, and the dependent variable was the distribution of membership across regions. Secondary Chi-Square analyses (McNemara’s test) were run to examine how the hub and Rich Club status of individual brain regions changed over time. Spearman rank correlations were conducted to examine the relationship between network metrics and drinking outcomes (daily average g/kg) during Free Access. Two-tailed tests and alpha of 0.05 were used for significance.

**Table 3:** Correlations between Baseline network metrics and Free-Access drinking levels.

	Connection frequency	Rich club coefficient	Rich club nodes	Rich club degree	Clustering coefficient	Assortativity
Free Access drinking	0.60	−0.26	0.71	−0.88^a^	−0.31	0.03

*Note. *n* = 6, Free-Access Drinking = average daily consumption in grams/kilogram during the 180 days of unrestricted access, ^a^*p* = 0.02*.

**Table 4:** The number of subjects for which each brain region was considered a hub of the network.

Brain region	Baseline	Free-Access
Parietal	3	0
Thalamus	4	2
Precuneus	2	1
Cerebellum	4	2
Amygdala	2	2
Hippocampus	2	4

*Nodes are considered hubs if they are in the upper 10% of the network for degree. Only nodes considered a hub for at least two subjects at any time point are presented. Note. n = 6*.

**Table 5:** The number of subjects for which each brain region was considered a member of the Rich Club.

Brain region	Baseline	Free-Access
Putamen	5	4
Hippocampus	5	5
Thalamus	5	3
Insula	5	2
Cerebellum	5	3
Parietal	4	3
Amygdala	4	5
OrbitoFrontal	4	3
Caudate	3	3
Precuneus	3	2

*Note. *n* = 6*.

## Results

Daily average g/kg EtOH consumption increased significantly once given free access [mean (SD); Free-Access = 4.7 (1.0), *p* < 0.01, Cohen’s *d* = 5.23], as shown in Table 1. Network metrics at Baseline and Free-Access (after 180 days unrestricted access) are shown in Table 2.

### Networks Predicting EtOH Consumption

**Figure 1:**
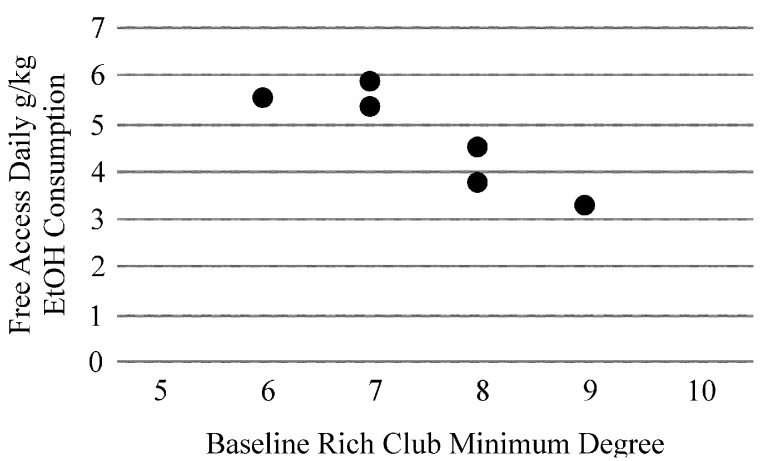
The minimum degree of the Rich Club of the alcohol naïve (Baseline) functional brain network was strongly related to future drinking when animals were provided free-access to alcohol (*r* = −0.88, *p* = 0.02).

Table 3 illustrates correlations between Baseline (EtOH naïve) network metrics and Free Access consumption. The minimum degree of the Rich Club at baseline was strongly related to Free Access consumption levels (Figure 1).

### Effects of EtOH on Networks

Table 2 shows mean and standard deviations of network metrics at Baseline and Free Access. The average connection frequency was significantly higher at Free Access than at Baseline, *t*_(5)_ = −3.20, *p* = 0.024, but no other differences in network metrics were observed.

### Specific Brain Regions

Table 4 demonstrates areas considered hubs across animals at each time point. For brain structures with multiple aspects, the region was considered a hub if any of the aspects were considered a hub (e.g., if either the posterior, medial, or anterior hippocampus was a hub, then Table 4 indicates the hippocampus as a hub). There was no significant change in brain regions considered hubs from Baseline to Free Access. Table 5 includes brain regions that were members of the Rich Club for at least three animals at any time point, again collapsing within regions. A significant decrease in the commonality of regions in the Rich Club across animals was observed from Baseline to Free Access, *t*_(9)_ = 2.74, *p* = 0.022. Tests of change in individual regions were not significant.

## Discussion

The current study demonstrates that characteristics of the Rich Club of alcohol-naïve brain networks are related to future drinking behaviors. In addition, following extended chronic drinking the connection strength in the network was altered; however, no effect on network metrics was observed. Finally, while hubs of the network were not observed to change significantly over time, membership in the Rich Club was significantly altered by chronic heavy drinking.

Aspects of *alcohol-naïve* resting-state functional brain networks were demonstrated to predict *future* drinking levels. Higher minimum degree of the Rich Club at Baseline (alcohol-naïve) was strongly correlated with decreased alcohol consumption during the future free access period. The Rich Club is a community of nodes within the network that have high degree and interconnectedness, serving as the “spine” of the network (Colizza et al., 2006). These nodes represent hubs within the network and communication among these nodes can often serve as “shortcuts” within the network, increasing efficiency of communication across otherwise distantly connected nodes. As the minimum degree of the Rich Club decreases, the centrality of Rich Club nodes also decreases, meaning fewer aspects of communication are routed through these nodes. Essentially, as the minimum degree decreases, the Rich Club becomes less differentiated from the other nodes in the network. This result suggests that the level of Rich Club differentiation may be predictive of future drinking levels, *even when measured prior to alcohol exposure*.

Alterations to the Rich Club subnetwork are likely to have broad and sweeping effects on brain communication and information processing (van den Heuvel and Sporns, 2013). Differences in Rich Club characteristics have been observed in many neurodevelopmental disorders, including schizophrenia (Collin et al., 2017), bipolar disorder (Wang et al., 2019), and autism (Hong et al., 2019). As such, the broad differences in Rich Club characteristics observed in this study are unlikely to serve as a direct “neurophenotype” of AUD without further refinement and empirical study. However, these results identify that differences in network topology are important to understanding individuals who might be at risk for future heavy drinking or AUD. Further, these results are consistent with previous work using the same NHP model indicating that premorbid behaviors and those occurring early in the drinking history may be predictive of future consumption levels. These factors include low cognitive flexibility (Shnitko et al., 2019), early drinking phenotypes (i.e., gulping vs. sipping; Grant et al., 2008; Baker et al., 2017), age, latency to begin drinking, and the number of “bouts” of drinking (Helms et al., 2014; Baker et al., 2017). The current results are the first to provide a brain-based factor indicative of future drinking in this model, suggesting that alcohol-naïve differences in Rich Club characteristics of functional brain networks also predict future drinking in this model.

These results extend recent findings in human participants demonstrating that white matter brain networks of individuals with AUD displayed lower Rich Club characteristics compared to their non-abusing siblings, who displayed lower levels compared to control participants (Zorlu et al., 2019). These results are also consistent with recent findings using the same NHP model indicating that chronic heavy drinking inhibits white matter growth (i.e., reduced connectivity) during late adolescence (Shnitko et al., 2019). While Zorlu et al. (2019) suggest potential premorbid differences in white matter network structure may be a marker of risk or susceptibility to AUD, the results of the current study provide direct empirical support for this hypothesis, showing that Rich Club characteristics of premorbid functional brain networks are directly related to future drinking levels. These results are also consistent with those of several longitudinal projects conducted in humans including the IMAGEN and NCANDA studies (Brumback et al., 2016; Maričić et al., 2020; Silveira et al., 2020). However, the current results examine functional RS brain networks and not reward based processing or structural aspects. The current study also did not acquire genetic or behavioral data as was acquired by IMAGEN, limiting further comparisons. Findings from the current study as well as these previous studies demonstrate that brain function and structure measured prior to exposure to alcohol may be able to identify individuals at risk for future heavy alcohol consumption.

Alcohol-induced changes in functional brain networks were observed following a period of exposure and free access to alcohol. The mean connection frequency increased following chronic heavy drinking; however, changes in network metrics were not observed. This suggests the general topology of networks were not altered (e.g., path lengths, clustering, etc.) Significant changes were seen in the membership of the Rich Club. There was much less consistency in Rich Club membership following chronic heavy drinking, suggesting the networks were being altered in an inconsistent manner across animals. It should be noted that the quantity of alcohol consumed during the free access period was fully determined by each animal. These results support the potential for a dose-dependent relationship between patterns of alcohol consumption and the effect on functional brain networks (Correas et al., 2015, 2016; López-Caneda et al., 2017; Perez-Ramirez et al., 2017).

Limitations of the current pilot study include the small sample size, which limits the complexity and sensitivity of analyses that can be conducted. Neuroimaging was conducted under anesthesia, which has known effects on brain function (Boveroux et al., 2010; Xie et al., 2013; Guldenmund et al., 2016). Possible interactions between the anesthetic and alcohol could have occurred, if not directly, then through the indirect development of tolerance. However, anesthesia was maintained at consistent levels and physiological indicators of arousal were monitored continuously, suggesting that levels of sedation were consistent across scans and animals. Additionally, after baseline data was acquired in the alcohol-naïve state, all animals entered the alcohol self-administration paradigm. We are mindful that this within-animal comparison across time limits insight into potential non-alcohol related changes over time or changes as a result of operant manipulations. However, test-retest scan sessions in monkeys 1 year apart and in humans 6 years apart (Stapleton-Kotloski et al., 2014) have established that networks remain stable over time.

Neuroimaging under conscious conditions will be required to completely understand the effects of alcohol on brain function using this model. The interval between ethanol access and MEG scans raises the possibility that some animals may have been experiencing symptoms of withdrawal (Winger and Woods, 1973). However, signs of withdrawal were not observed during the same time periods on non-imaging days (Pieper and Skeen, 1977; Cuzon Carlson et al., 2011). Also, propofol was used as the anesthetic agent, helping to ensure animals were not experiencing withdrawal symptoms *during* scans (Shin et al., 2018). Finally, animals who ceased alcohol consumption prior to scans did so voluntarily and in a time frame consistent with non-imaging days and were not forcibly fasted.

## Conclusions

The current study identified a relationship between functional brain networks in the alcohol-naïve state and future alcohol consumption, consistent with other work using this model demonstrating early behavioral markers of future drinking. This is the first brain-based predictor of future alcohol consumption identified for this model. Additionally, significant alteration in the Rich Club of the network was observed following 180 days of chronic heavy consumption of alcohol, an earlier time point than examined in previous works. Future work will be invaluable in clarifying the changes, and specifying the timing of those changes, that infer risk specific to AUD in humans.

## Data Availability Statement

The raw data supporting the conclusions of this article will be made available by the authors, without undue reservation.

## Ethics Statement

The animal study was reviewed and approved by Wake Forest School of Medicine ACUC.

## Author Contributions

JR, JS-K, AD, DG, and JD contributed to the study design, JR, JS-K, AD, GA, PE, and JD contributed to data acquisition. JR, JS-K, DG, GA, and JD contributed to data analysis and interpretation. JR, JS-K, GA, AD, PE, DG, and JD contributed to manuscript preparation. All authors contributed to the article and approved the submitted version.

## Conflict of Interest

The authors declare that the research was conducted in the absence of any commercial or financial relationships that could be construed as a potential conflict of interest.
